# Supplementary material for: Fungal dissemination is limited by liver macrophage filtration of the blood
Source: Nat Commun. 2019 Oct 8;10:4566. doi: 10.1038/s41467-019-12381-5 (PMC6783440; doi:10.1038/s41467-019-12381-5)
Supplement: Supplementary file 1 — Supplementary information [file 41467_2019_12381_MOESM1_ESM.pdf]

Fungal dissemination is limited by liver macrophage filtration of the blood

Sun et al.

Supplementary Information

Supplementary Figure 1

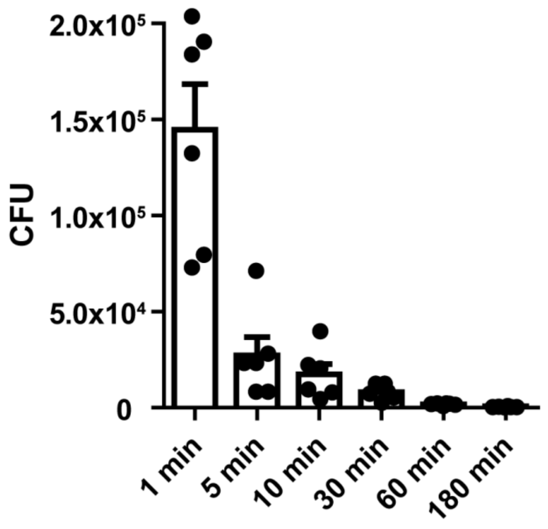

Supplementary Figure 1. Kinetics of blood CFU in mice infected with *C. neoformans*.

Mice (n=6 mice) were i.v. infected with $5x10^6$ *C. neoformans* H99. The fungal burden in the blood collected from the tail vein was determined at various time points post infection. Data are expressed as mean ± SEM. Data are from biologically distinct samples. Source data are provided as a Source Data file.

Supplementary Figure 2

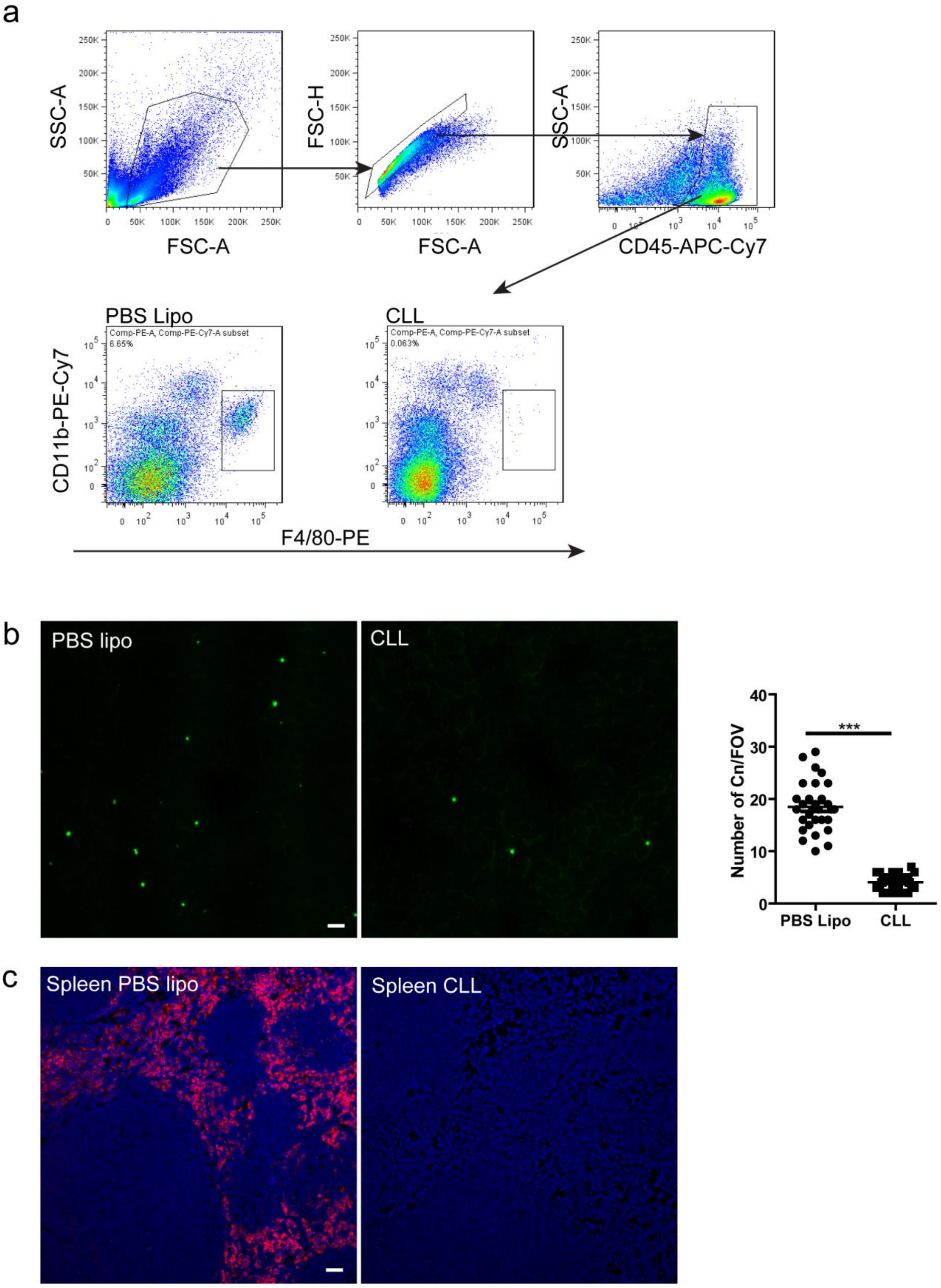

Supplementary Figure 2. Depletion of Kupffer cells (KCs) by clodronate liposomes (CLL) leads to reduced capture of *C. neoformans* in the liver.

(**a**) Representative flow cytometry plots showing the depletion of KCs in the liver of mice 24 h after i.v. injection with 200 µl CLL. Control mice were i.v. injected with PBS liposomes. The gating strategy is shown above (KCs are defined as $CD45^{+}F4/80^{+}CD11b^{int}$ cells). The same gating strategy for KCs was also used for Fig. 4b, 5c, 6h and Supplementary Fig. 5e. (**b**) The number of *C. neoformans* captured in the liver after depletion of KCs by CLL. Mice (n=5 mice/group) were i.v. injected with 200 µl CLL or PBS liposomes as control and 24 h later i.v. infected with $20x10^{6}$ *C. neoformans* H99 labeled with Uvitex 2B (green, pseudocolor). The mice were euthanized 3 h post infection; the liver was removed and frozen in OCT compound. The number of *C. neoformans* in frozen sections (7 µm) was enumerated by fluorescence microscopy. Left panel: representative images; right panel: quantification as per field of view (FOV, 0.5 mm x 0.5 mm). (**c**) The depletion of spleen macrophages by CLL treatment. Mice were i.v. injected with 200 µl CLL or PBS liposomes as control. Spleen tissues were collected 24 h after the treatment and frozen in OCT compound. The tissues were cut into 7 µm slices and stained by AF647 conjugated anti-mouse F4/80 mAb to detect macrophages (red, pseudocolor), nuclei were stained by DAPI (blue). Scale bars: 25 µm. Data expressed as mean ± SEM are from different field of views of mice pooled together. ***p<0.001 by unpaired two-tailed Student's *t* test. Source data are provided as a Source Data file.

Supplementary Figure 3

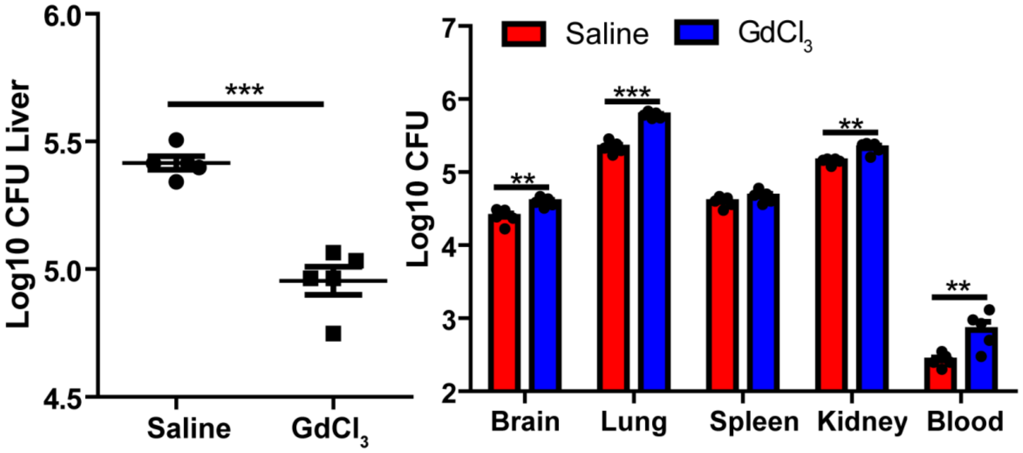

Supplementary Figure 3. Treatment of mice with gadolinium chloride ($GdCl_3$) reduces liver capture of *C. neoformans*.

Mice (n=5 mice/group) were i.v. injected with $GdCl_3$ (60 mg/kg) in saline or saline alone as control. Twenty-four hours later, mice were i.v. infected with $5x10^6$ *C. neoformans* H99. The fungal burdens were determined in the liver (left panel) and other tissues (right panel) 3 h after infection. Data are from biologically distinct samples expressed as mean ± SEM from 1 of 3 independent experiments. $^{**}p<0.01$, $^{***}p<0.001$ by unpaired two-tailed Student's *t* test. Source data are provided as a Source Data file.

Supplementary Figure 4

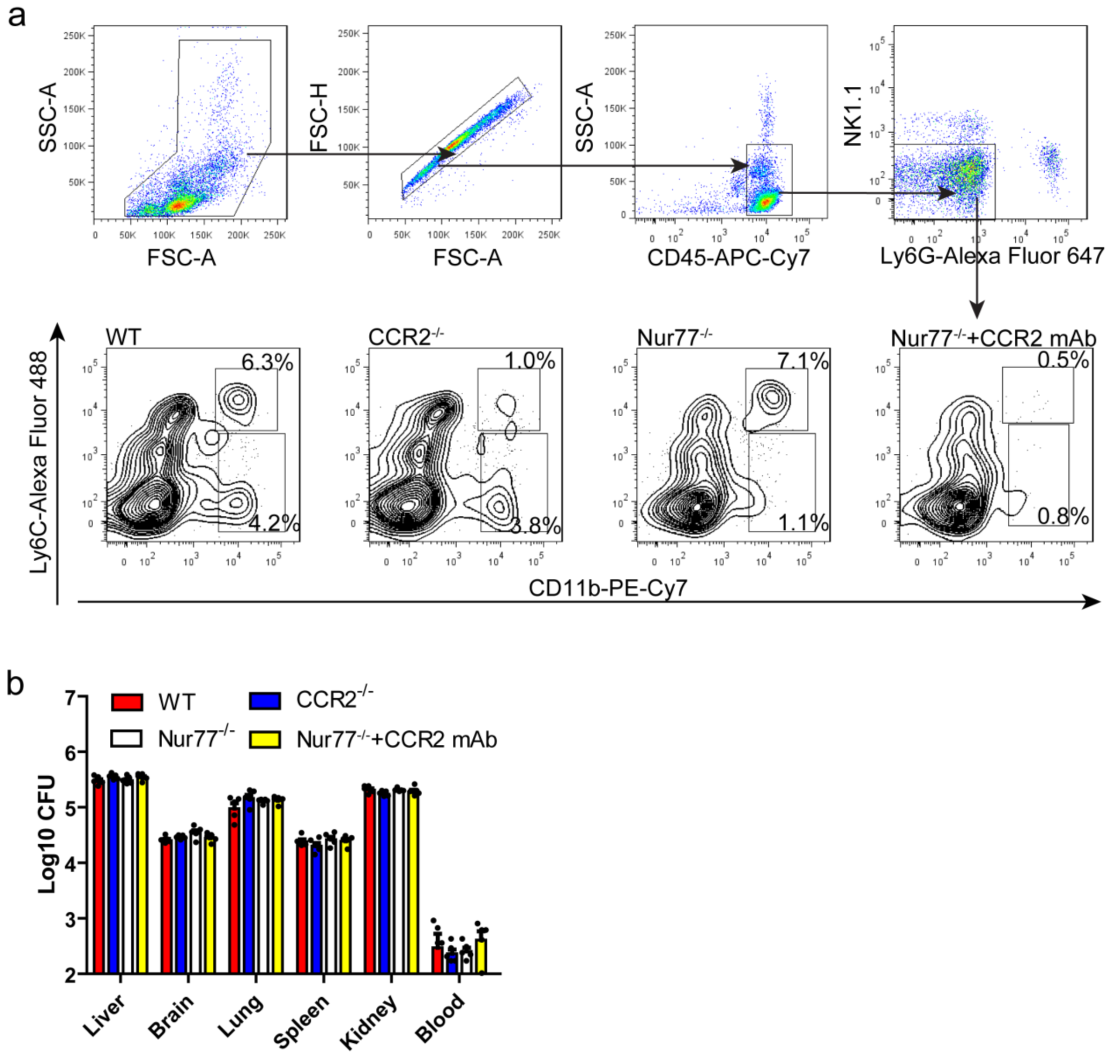

Supplementary Figure 4. Loss of circulating monocytes does not affect the capture of *C. neoformans* in the liver.

(**a**) Representative flow cytometry plots showing the frequencies of Ly6C^{hi} and Ly6C^{low} subsets of monocytes in the blood of WT mice, CCR2$^{-/-}$ mice, Nur77$^{-/-}$ mice, and Nur77$^{-/-}$ mice treated (i.v. injection) with 6 μg of anti-CCR2 antibody (MAB55381, R&D systems) to deplete Ly6C^{hi} monocytes. The gating strategy is shown above. (**b**) WT mice, CCR2$^{-/-}$ mice, Nur77$^{-/-}$ mice, and Nur77$^{-/-}$ mice treated with anti-CCR2 antibody (n=5 mice/group) were i.v. infected with 5×10^6 *C. neoformans* H99; the CFU in the liver, blood, and other organs were enumerated 3 h

after infection. Data are from biologically distinct samples expressed as mean ± SEM. Not significant by two-way ANOVA. Source data are provided as a Source Data file.

Supplementary Figure 5

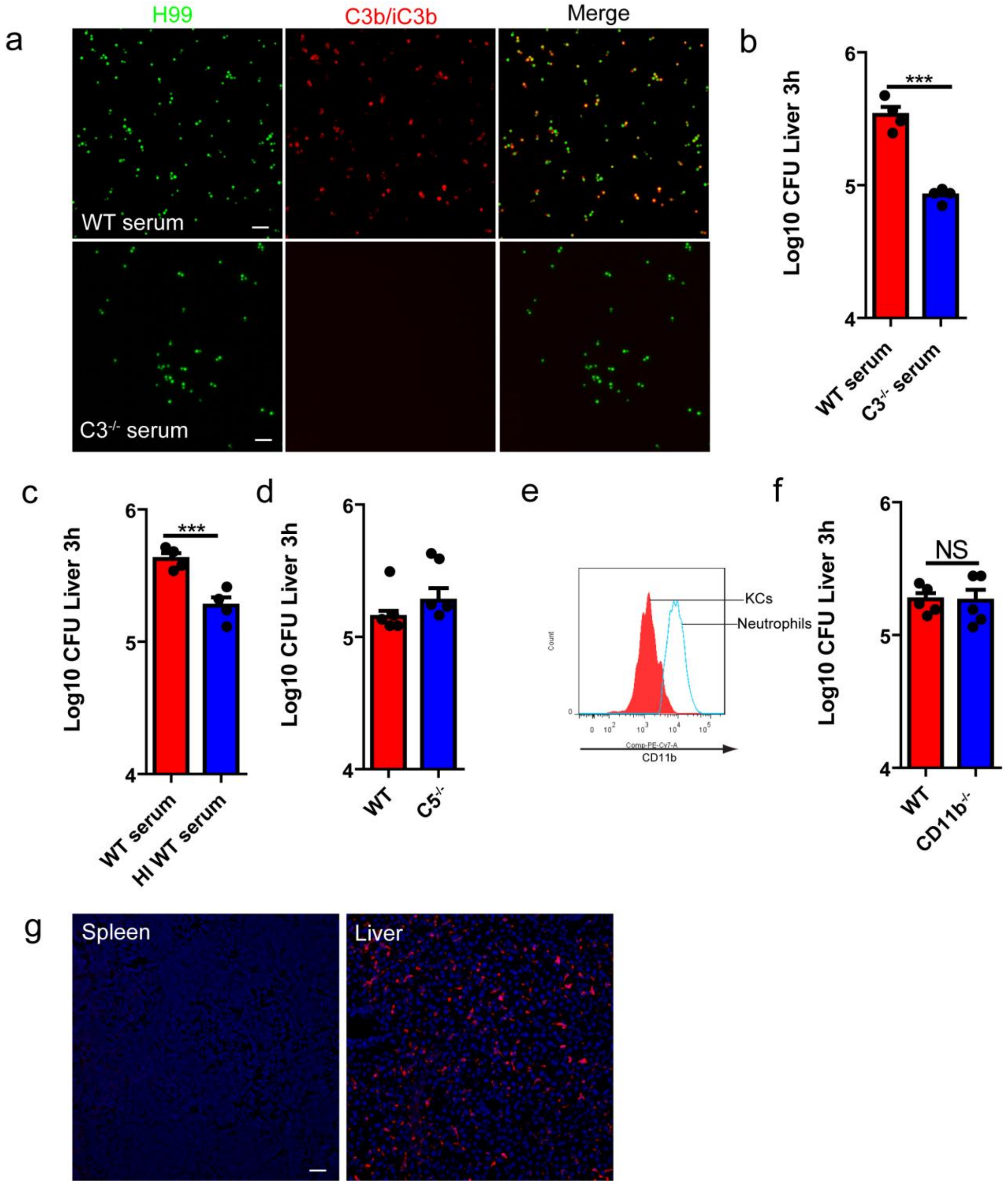

Supplementary Figure 5. C3, but not C5 or CR3, is required for liver capture of *C. neoformans*.

(**a**) GFP-labeled *C. neoformans* H99 was incubated with fresh WT or C3$^{-/-}$ serum at 37°C for 15 min to detect complement deposition on the yeast cells. Green, *C. neoformans*; red, C3b/iC3b. (**b**) C3$^{-/-}$ mice (n=4 mice/group) were i.v. infected with 5x10^{6} *C. neoformans* H99 incubated with WT or C3$^{-/-}$ serum. Liver CFU was

enumerated 3 h after infection. (**c**) $C3^{-/-}$ mice (n=4 mice/group) were i.v. infected with $5x10^6$ *C. neoformans* H99 incubated with WT or heat-inactivated (HI) serum. Liver CFU was enumerated 3 h after infection. (**d**) WT and $C5^{-/-}$ mice (n=5 mice/group) were i.v. infected with $5x10^6$ *C. neoformans* H99. Liver CFU was enumerated 3 h after infection. (**e**) Comparison of CD11b expression on neutrophils ($CD45^+Ly6G^+$) and liver KCs. (**f**) WT and $CD11b^{-/-}$ (CR3 deficient) mice (n=5 mice/group) were i.v. infected with $5x10^6$ *C. neoformans* H99. Liver CFU was enumerated 3 h after infection. (**g**) The detection of CRIg expression in the spleen (left) and liver (right). The spleen and liver from naïve mice were frozen in OCT compound and cut into 7 μm thick slices. Immunofluorescence staining was performed using anti-mouse CRIg primary mAb and fluorescent secondary mAb (red). The nuclei were stained by DAPI (blue). Scale bars: 25 μm. Data are from biologically distinct samples expressed as mean ± SEM from 1 of 3 independent experiments. NS: not significant, ***p<0.001 by unpaired two-tailed Student's *t* test. Source data are provided as a Source Data file.

Supplementary Figure 6

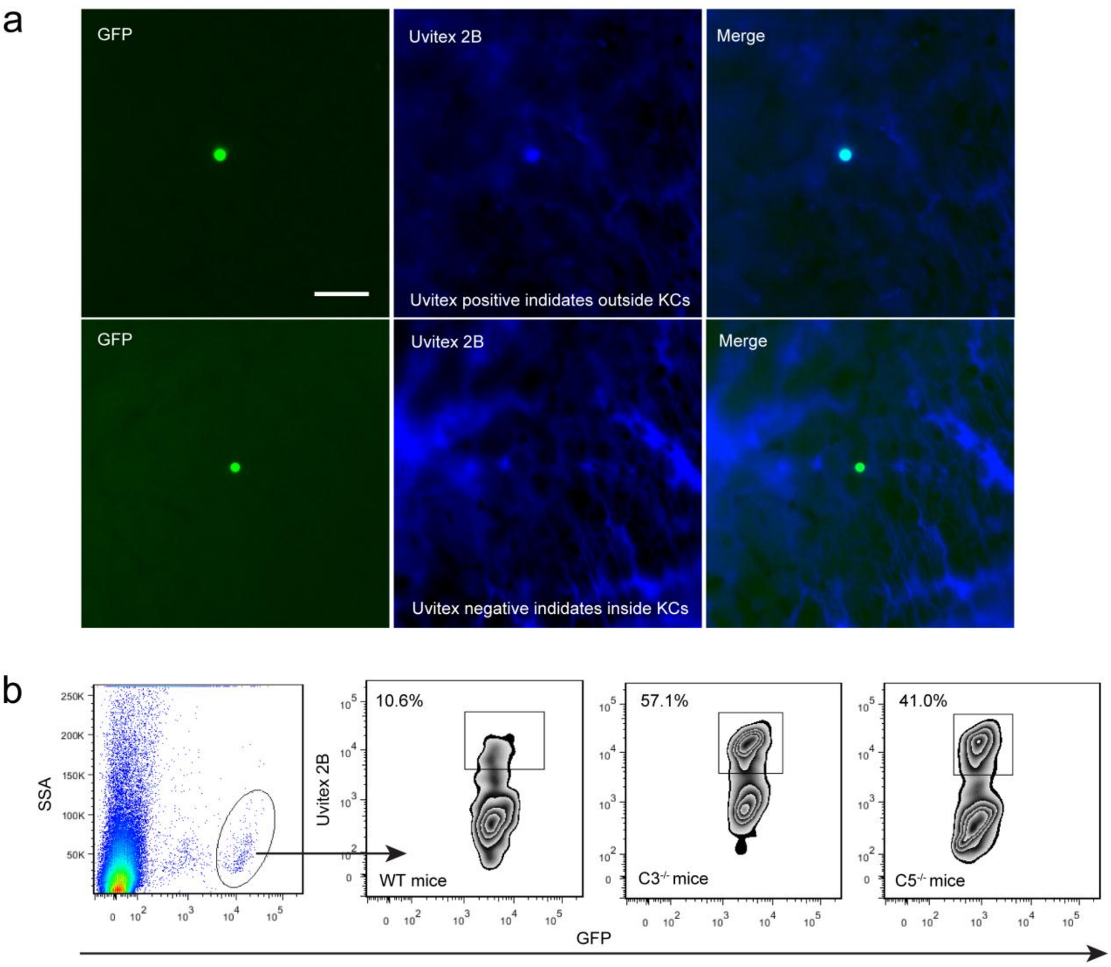

Supplementary Figure 6. Reduced phagocytosis of *C. neoformans* in the liver of C3$^{-/-}$ and C5$^{-/-}$ mice as determined by in vivo Uvitex 2B staining.

(**a**) Mice were i.v. infected with 20x10^{6} GFP-labeled *C. neoformans* H99; the mice were i.v. injected with 100 μl Uvitex 2B (1% w/v in PBS) 15 min post infection. The mice were euthanized and perfused 2 min after Uvitex 2B injection; the liver was excised and examined under fluorescence microscope. The yeast cells labeled by Uvitex 2B (blue, pseudocolor) were outside phagocytes, while those without Uvitex 2B staining were inside phagocytes. Scale bar: 25 μm. (**b**) Representative flow cytometry plots showing the percentage of unphagocytosed yeast cells (Uvitex 2B positive) in the liver of WT, C3$^{-/-}$ and C5$^{-/-}$ mice 15 min post infection with 20x10^{6} GFP-labeled *C. neoformans* H99.

Supplementary Figure 7

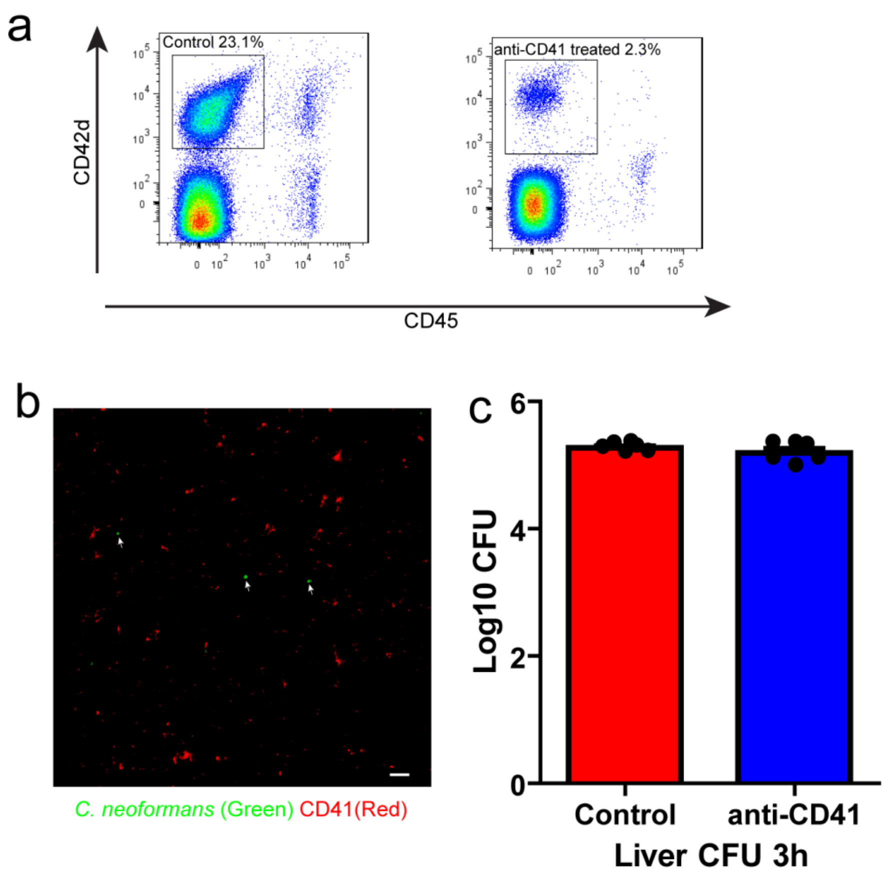

Supplementary Figure 7. Depletion of platelets does not affect liver capture of *C. neoformans*.

(**a**) Mice were i.v. injected with 100 μg anti-CD41 antibody (Catalog No. 133910, Biolegend) to deplete platelets; 24 h later, the depletion of platelets was confirmed by flow cytometry. (**b**) Immunofluorescence staining of frozen liver sections showed that platelets (red) did not bind to *C. neoformans* (arrows, green) in the liver of mice 30 min after i.v. infection with 20×10^6 GFP-labeled *C. neoformans* H99. (**c**) No significant difference was detected in the liver fungal burdens in mice treated with anti-CD41 antibody and mice treated with control antibody (n=6 mice/group) 3 h after i.v. infection with 5×10^6 *C. neoformans* H99. Scale bar: 25 μm. Data are from biologically distinct samples expressed as mean ± SEM. Not significant by unpaired two-tailed Student's *t* test. Source data are provided as a Source Data file.

Supplementary Figure 8

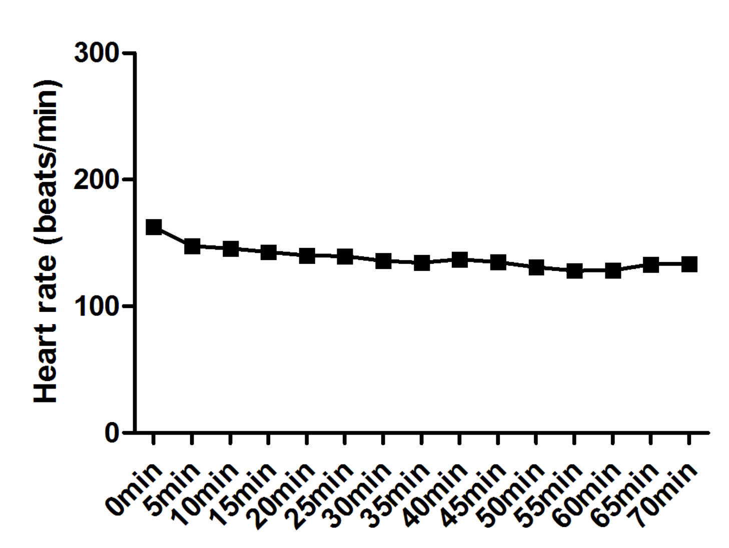

Supplementary Figure 8. Heart rate of mice during experimental procedure. Mice (n=5 mice) were anesthetized by i.p. injection of a mixture of 200 mg/kg ketamine hydrochloride and 10 mg/kg xylazine. The liver surgery was performed and mice were mounted on the imaging stage for intravital imaging. 30 min after the surgery, mice were i.v. injected with an additional 50 μl of mixture containing ketamine (20 mg/ml) and xylazine (1 mg/ml). The heart rate was measured during intravital imaging using MouseSTAT (Kent Scientific, USA). Data are from biologically distinct samples expressed as mean ± SEM. Source data are provided as a Source Data file.
