# Supplementary material for: Fungal dissemination is limited by liver macrophage filtration of the blood
Source: Nat Commun. 2019 Oct 8;10:4566. doi: 10.1038/s41467-019-12381-5 (PMC6783440; doi:10.1038/s41467-019-12381-5)
Supplement: Supplementary file 3 — Description of Additional Supplementary Files [file 41467_2019_12381_MOESM3_ESM.docx]

**Description of Additional Supplementary Files**

**File Name: Supplementary Movie 1**

**Description:** Video taken by IVM showing a sudden stop of C. neoformans in the liver of mice after i.v. injection of GFP-labeled C. neoformans H99. Some of the yeast cells were released back to the bloodstream following trapping.

**File Name: Supplementary Movie 2**

**Description:** Video taken by IVM showing the behavior of C. neoformans in the liver of mice treated with PBS liposomes (control; left panel) or clodronate liposomes (to deplete KCs; right panel) after i.v. injection of GFP-labeled C. neoformans H99.

**File Name: Supplementary Movie 3**

**Description:** Video taken by IVM showing the trapping and release of C. neoformans in the liver of C3-/- mice after i.v. injection of GFP-labeled C. neoformans H99.

**File Name: Supplementary Movie 4**

**Description:** A 3D reconstructive movie of Figure 5A showing phagocytosis of C.

neoformans (green) by KCs (red).
